# circFOXP1 Promotes Pancreatic Ductal Adenocarcinoma Progression Through Regulating EREG/MAPK/ERK Axis

**DOI:** 10.1111/jcmm.71230

**Published:** 2026-07-06

**Authors:** Leyi Huang, Dan Su, Rihua He, Bingzheng Zhong, Qiang Wang, Fuxu Feng, Jingwen Li, Jie Cao, Quanbo Zhou, Xiaofeng Guo

**Affiliations:** ^1^ Department of Pancreas Center, Guangdong Institute of Cardiovascular Diseases Guangdong Provincial People's Hospital (Guangdong Academy of Medical Sciences), Southern Medical University Guangzhou Guangdong People's Republic of China; ^2^ Department of Pancreatobiliary Surgery Sun Yat‐Sen Memorial Hospital, Sun Yat‐Sen University Guangzhou Guangdong People's Republic of China; ^3^ Guangdong Provincial Key Laboratory of Malignant Tumor Epigenetics and Gene Regulation Medical Research Center Sun Yat‐Sen Memorial Hospital, Sun Yat‐Sen University Guangzhou Guangdong People's Republic of China; ^4^ Department of General Surgery Peking Union Medical College Hospital, Chinese Academy of Medical Sciences and Peking Union Medical College Beijing People's Republic of China; ^5^ Department of Gastrointestinal, Hernia, and Abdominal Wall Surgery Guangzhou First People's Hospital, South China University of Technology Guangzhou Guangdong People's Republic of China; ^6^ Zhongshan School of Medicine Sun Yat‐Sen University Guangzhou Guangdong People's Republic of China; ^7^ Lead Contact Guangzhou People's Republic of China

**Keywords:** circFOXP1, epiregulin, miR‐320b, pancreatic ductal adenocarcinoma

## Abstract

Circular RNAs (circRNAs) are indispensable for triggering pancreatic ductal adenocarcinoma (PDAC) progression. However, the specific biological processes and mechanisms by which circRNAs influence PDAC remain largely unknown. Here, we reported that circFOXP1 is a critical promoter of PDAC progression, exhibiting marked upregulation in patient tumour tissues that correlates with advanced TNM stage and poor prognosis. Functional studies demonstrate that circFOXP1 knockdown significantly suppresses PDAC cell proliferation, migration, and invasion in vitro and in vivo. Mechanistically, circFOXP1 acts as a molecular sponge for miR‐320b, leading to the upregulation of epidermal growth factor receptor (EGFR) ligand Epiregulin (EREG) and the subsequent activation of the MAPK/ERK signalling pathway, which is crucial for maintaining the aggressive phenotype of PDAC. The blockade of EREG using neutralizing antibodies in vivo substantially abrogates circFOXP1‐induced tumorigenesis. Our findings underscore the potential of circFOXP1 as a novel biomarker and propose a novel therapeutic target to improve survival in PDAC patients.

**Abbreviations**
ANOVA: analyses of variance
CCK‐8: cell counting kit 8
circRNAs: circular RNAs
DFS: disease‐free survival
EdU: 5‐Ethynyl‐2′‐deoxyuridine
EGFR: epidermal growth factor receptor
ERK: extracellular regulated protein kinases
FISH: fluorescence in situ hybridization
HE: haematoxylin and eosin
IHC: immunohistochemistry
MAPK: mitogen‐activated protein kinase
mut: mutant
NATs: normal adjacent tissues
OS: overall survival
PBS: phosphate‐buffered saline
PDAC: pancreatic ductal adenocarcinoma
RBPs: RNA‐binding proteins
qRT‐PCR: quantitative real‐time PCR
SCID: severe combined immunodeficient
TNM: tumour‐node‐metastasis
WT: wild‐type

## 1 Background

Pancreatic ductal adenocarcinoma (PDAC) is an aggressive and clinically challenging malignancy within the gastrointestinal tract. Despite significant advances in recent decades that have greatly broadened our understanding of the aetiology, biology and pathogenesis of PDAC, the overall survival of patients with PDAC remains dismally low at approximately 13%. This is mainly because a large proportion of patients are diagnosed only after tumour cells have invaded neighbouring organs or metastasized to distant organs [1, 2]. Also, PDAC exhibits inherent or acquired resistance to both conventional chemotherapy and emerging immunotherapy [3]. This underscores the urgent need for improved diagnostic and predictive tools, such as image‐based technologies and biomarkers, to precisely differentiate PDAC patients from their tumour‐free individuals at earlier stages and develop effective treatment for PDAC patients.

Circular RNAs (circRNAs) are a distinct type of covalently closed transcripts generated through the back‐splicing of pre‐mRNA. Unlike linear RNAs, circRNAs lack polyadenylated tails and 5′‐3′ polarity, rendering them higher resistance to exonucleases [4]. In recent years, emerging evidence has highlighted the crucial regulatory roles of circRNAs in various human diseases, including neurological disorders, cardiovascular diseases and multiple types of cancer [5, 6]. The mechanisms by which circRNAs exert their physiological and pathological effects include serving as miRNA sponges for post‐transcriptional regulation of gene expression, directly interacting with RNA‐binding proteins (RBPs) to modulate their subcellular location or function, regulating transcription and mRNA translation, and functioning as templates for the translation of novel protein isoforms [7]. In PDAC, circRNAs have been implicated in multiple aspects including tumour growth, invasion, metastasis and chemotherapy resistance [8, 9, 10]. However, the specific roles of circRNAs in regulating key oncogenic pathways in PDAC, such as the Epidermal growth factor receptor (EGFR) signalling cascade, remain largely unexplored.

EGFR, also known as ERBB1, is frequently overexpressed in Kirsten rat sarcoma viral oncogene homologue (Kras) mutation‐driven malignancies, such as lung, colorectal and pancreatic cancers [11]. Epiregulin (EREG), a ligand of EGFR, exhibits a low level in normal versus tumour tissues. EREG is overexpressed in various tumours, contributing to their initiation and progression primarily through binding to EGFR. This interaction activates downstream signalling cascades including phosphatidylinositol‐4,5‐bisphosphate 3‐kinase (PI3K)/AKT, mitogen‐activated protein kinase (MEK)/extracellular signal‐regulated kinase (ERK) and signal transducer and activator of transcription (STAT) pathways [12, 13]. Recent studies have revealed additional roles of EREG in cancer, with emerging evidence suggesting that EREG originating from macrophages can drive resistance to EGFR‐tyrosine kinase inhibitors (EGFR‐TKI) [14]. In PDAC, EREG has been found to be upregulated in tumour tissues compared to normal pancreas, with the potential to promote cancer cell growth in vitro [15]. Yet, it remains unknown whether circRNAs are implicated in the regulation of EREG expression and its downstream signalling pathways in PDAC.

In our study, we identified a novel circular RNA, circFOXP1 (hsa_circ_0001320), which is aberrantly upregulated in PDAC tissues compared to adjacent tissue. We found that patients with higher circFOXP1 expression in PDAC tissues correlate with worse prognosis, suggesting its potential as a prognostic biomarker. Our further experiments demonstrated that circFOXP1 promotes PDAC cell proliferation, migration, and invasion in vitro, as well as sustained tumour growth and metastasis in vivo. Mechanistically, we uncovered that circFOXP1 functions as a sponge for miR‐320b, leading to elevated EREG expression, thereby activating the downstream MAPK/ERK pathway to maintain the aggressive phenotype of PDAC. Our findings not only elucidate a novel circRNA‐miRNA‐mRNA regulatory axis in PDAC pathogenesis but also provide compelling evidence for circFOXP1 as both a promising prognostic biomarker and a potential therapeutic target in PDAC. Overall, our finding highlights the crucial role of the circFOXP1/miR‐320b/EREG axis in PDAC and identifies circFOXP1 as a promising prognostic biomarker and therapeutic target for PDAC.

## 2 Materials and Methods

### 2.1 Cell Culture

The human pancreatic ductal cell line hTERT‐HPNE and various human PDAC cell lines (AsPC‐1, BxPC‐3, CFPAC‐1 and PANC‐1) were obtained from the American Type Culture Collection (ATCC, Rockville, MD, USA). These cells were cultured in a humidified incubator at 37°C with 5% CO_2_, utilizing Dulbecco's modified Eagle's medium (DMEM), RPMI 1640 medium, or Iscove's Modified Dulbecco's medium (IMDM) from Gibco in a humidified incubator with 5% CO_2_. Fetal bovine serum and penicillin/streptomycin were supplemented in the medium to achieve final concentrations of 10% and 1%, respectively.

### 2.2 Cell Transfection

For transient knockdown or overexpression, PDAC cells at 70% confluence were transfected with small interfering RNAs (siRNAs) using Lipofectamine RNAi Max (Invitrogen, USA) or plasmids using Lipofectamine 3000 reagent (Invitrogen, USA) according to the manufacturer's instructions. siRNA oligonucleotides were purchased from IGE (Guangzhou, China). Full‐length circFOXP1 sequence was cloned into the pCD‐ciR vector. For lentivirus production, HEK 293 T cells were transfected with the indicated plasmids, psPAX2 and pMD2.G. PDAC cells were infected with lentivirus‐containing supernatants in the presence of 5 μg/mL polybrene. Infected cells were selected with puromycin. Oligonucleotide sequences are in Table S1.

### 2.3 RNA Isolation and qRT‐PCR

Total RNA was extracted from human tissues and cell lines using RNAiso Plus (Takara, Japan). RNA was reverse‐transcribed to cDNA using PrimeScript RT Reagent Kit (Takara, Japan). Quantitative real‐time PCR (qRT‐PCR) was performed using TB Green Premix Ex TaqTM kit (Takara, Japan) on a Light Cycler 480 Detection System (Roche, Switzerland). Transcript expression was analysed using the 2^−ΔΔCt^ method, with U6 small nuclear RNA (snRNA) as the internal control and GAPDH (glyceraldehyde‐3‐phosphate dehydrogenase) as the target transcript. Primers are listed in Table S2.

### 2.4 RNase R Treatment and Actinomycin D Assay

To test the stability of circRNAs or linear mRNAs, 0.2 μL RNase R (Beyotime, China) or an equal volume of DNase and RNase‐free water was added to 5 μg of total RNA, followed by incubation at 37°C for 15 min. For actinomycin D assay, cells were treated with 2 mg/L actinomycin D (Selleck, USA) for 4 h, 8 h, 12 h or 24 h. DMSO was used as the negative control. After treatment, total RNA was isolated and subjected to qRT‐PCR to monitor gene expression.

### 2.5 Western Blotting

Whole‐cell extracts of cultured cells were obtained using RIPA lysis buffer (CWBIO, China) with protease‐ and phosphatase‐inhibitors (CWBIO, China). Proteins (20–40 μg) were separated by sodium dodecyl sulphate polyacrylamide gel electrophoresis (SDS‐PAGE) and then transferred to polyvinylidene fluoride (PVDF) membranes. Membranes were blocked with 5% bovine serum albumin (BSA) in Tris‐buffered saline‐Tween 20 (TBST) at room temperature, incubated with primary antibodies overnight at 4°C, then with horseradish peroxidase (HRP)–linked secondary antibodies for 1 h. Images were captured using an ECL detection system (Biorad, USA). All primary antibodies used are summarized in Table S3. All uncropped images are shown in Appendix S2.

### 2.6 Subcellular Fractionation

Nuclear and cytoplasmic fractions were isolated using the PARIS kit (Life Technologies, USA). RNA was extracted from each fraction, and the subcellular distribution of transcripts was determined by qRT‐PCR, using U6 snRNA and GAPDH as nuclear control and cytoplasmic control, respectively.

### 2.7 Fluorescence In Situ Hybridization (FISH)

The subcellular localization of circFOXP1 and miR‐320b was visualized using an In Situ Hybridization Kit (Gene Pharma, China) following the standard protocol. In brief, cells were fixed with 4% paraformaldehyde (PFA), pre‐hybridized with the provided buffer solution and incubated with Cy3‐labelled circFOXP1 or FAM‐labelled miR‐320b probes overnight at 37°C. Images were captured using a ZEISS confocal microscope (Carl Zeiss AG, Germany). Probe sequences are listed in Table S4.

### 2.8 Wound Healing Assay

PDAC cells seeded in six‐well plates at over 90% confluence were scratched with 200 μL sterile pipette tips. After washing with PBS, cells were cultured in serum‐free medium to minimize the effect of cell proliferation. Images were captured using an Olympus IX71 digital camera (Center Valley, PA, USA) and analysed with the Image J software (NIH, USA).

### 2.9 Transwell Assays

Transwell migration and invasion assays were performed to evaluate the migratory and invasive abilities of pancreatic cancer cells. Transfected cells suspended in 200 μL of FBS‐free medium were added to the upper chamber, uncoated for migration or Matrigel‐coated for invasion. The lower chamber contained 500 μL of medium with 1% FBS. After 24 h incubation, migrated or invaded cells were fixed in 4% PFA and stained with 0.1% crystal violet. Images were captured using a Nikon NI‐U digital camera (Japan). Migrated or invaded cell counts were quantified using the Image J software (NIH, USA).

### 2.10 EdU Incorporation Assay

Transfected cells were incubated in six‐well plates with 20 mM 5‐Ethynyl‐2′‐deoxyuridine (EdU) for 2 h (PANC‐1, BxPC‐3) or 4 h (CFPAC‐1). Cells were then fixed with 4% PFA, permeabilized with 0.3% Triton X‐100/PBS, blocked with 3% BSA/PBS and labelled using Alexa Fluor dyes using the Beyo Click EdU‐488 (or 555) detection kit (Beyotime, China). All images were captured with a fluorescent microscope.

### 2.11 Colony Formation Assay

A total of 500 PDAC cells were seeded in six‐well plates and cultured at 37°C for 2 weeks. Then, cells were fixed with 4% PFA at RT for 20 min, followed by staining with 0.1% crystal violet at RT for 20 min. Experiments were performed in triplicate for all groups, and visible colonies were manually counted.

### 2.12 Apoptosis Assay

The Annexin V‐FITC Apoptosis Detection Kit (YEASEN, Shanghai, China) was applied to detect cell apoptosis according to the manufacturers' instructions. In brief, transfected PDAC cells were trypsinized, washed with PBS, and stained with Annexin V‐FITC and PI. After incubation for 30 min, cells were loaded onto a CytoFLEX flow cytometer (Beckman Coulter, USA), and the results were analysed with the Flowjo software (USA).

### 2.13 RNA Pull‐Down Assay

Biotin‐labelled circFOXP1 junction‐specific and scramble probes were synthesized by GenePharma (Suzhou, China). The sequences of probes are listed in Table S5. Before lysing cells, the biotinylated probe and oligo probe were incubated with streptavidin‐coated magnetic beads (Invitrogen, Waltham, MA, USA) at 4°C for 4 h. A total of 2 × 10^7^ PDAC cells were lysed in buffer containing 0.02 M Tris–HCl (pH 7.5), 0.1 M KCl, 5 mM MgCl_2_, 0.5 mM DTT, 0.5% NP‐40, 60 U/mL RNase inhibitor (Promega), 1× protease inhibitor EDTA‐free (05892791001, Sigma, USA) on ice for 20 min, followed by centrifuging at 4°C for 30 min. Then, the supernatants were mixed with magnetic beads overnight at 4°C to capture RNA–RNA complex, which was later eluted from the beads with elution buffer (0.1% SDS, 2 mM EDTA, 10 mM Tris–HCl pH 8.0, 150 mM NaCl). RNAiso Plus (Takara, Japan) was added for RNA isolation and qRT‐PCR analysis was performed to screen for circFOXP1‐interacting miRNAs.

### 2.14 Biotin‐Labelled miRNA Capture

To confirm the interaction between circFOXP1 and miR‐320b, biotin‐labelled wild‐type (WT) or mutant miRNA mimics were transfected into PDAC cells using Lipofectamine 3000 reagent (Invitrogen, USA), and cells were allowed to grow for 48 h. Then, cells were lysed and the supernatants were incubated with pre‐blocked streptavidin‐coated magnetic beads at 4°C overnight. After elution, RNA was isolated for subsequent qRT‐PCR analysis.

### 2.15 Dual Luciferase Reporter Assay

The circFOXP1 wild‐type or mutant plasmid, luciferase reporter plasmid, and miRNA mimics were simultaneously transfected into HEK‐293 T cells using Lipofectamine 3000 reagent (Invitrogen, USA). Twenty‐four hours later, the transfected cells were seeded onto 96‐well plates, and luciferase activities were determined by a dual‐luciferase reporter kit (Promega, USA) as indicated by the manufacturer.

### 2.16 Immunohistochemistry (IHC)

Formalin‐fixed, paraffin‐embedded tissues were deparaffinized and hydrated, after which heat‐mediated antigen retrieval was performed using a pressure cooker. The sections were then blocked with 3% H_2_O_2_ and incubated with primary antibodies at 4°C overnight. A REAL EnVision Detection Kit (HRP‐linked, K5007, Dako) was used to reveal the locations of the antigens followed by counterstaining with haematoxylin.

### 2.17 Animal Experiments

All animal experiments in this study were approved by the IACUC, Jennio Biotech Co. Ltd. The subcutaneous xenograft model was applied to evaluate the effect of circFOXP1 knockdown or overexpression on in vivo growth of PDAC cells. A total of 3 × 10^6^ stably transfected cells were subcutaneously injected into the left hind flank of BALB/c nude mice aged 4 weeks. Every week, the maximum (length) or minimum (width) diameter of the subcutaneous tumour was measured and document data was used to calculate tumour volume using the following formula: volume = (width^2^ × length)/2. Five weeks later, the mice were sacrificed and tumour tissues were excised, weighed, fixed, embedded with paraffin and sliced for HE (haematoxylin & Eosin) or IHC staining.

For in vivo metastasis assay, 2 × 10^6^ PDAC cells resuspended with FBS‐free medium were injected into the lateral tail vein of severe combined immunodeficiency (SCID) mice using insulin needles. The mice were kept in a pathogen‐free environment for 5 weeks, after which they were injected i.p. with VivoGlo luciferin (150 mg/kg, Promega, USA) and anaesthetised using isoflurane inhalation. Ten minutes after injection, a Bruker In Vivo Xtreme imaging system (Bruker, Karlsruhe, Germany) was used for luminescence detection. Then, all mice were euthanized and their lungs were excised, photographed and assessed for metastasis via H&E staining.

### 2.18 Microarray Analysis

CircRNA microarray is manufactured by Arraystar Technologies (Rockville, MD, USA). Five pairs of PDAC tissues and NAT tissues were used to isolate total RNAs, which were subsequently subjected to microarray sequencing. Differentially expressed circRNAs with statistical significance between groups were identified using the following criteria: log_2_ (fold change) ≥ 1 and *p* < 0.05. The microarray analysis work was performed by Bo Hao Bio‐tech (Guangzhou, People's Republic of China).

### 2.19 RNA Sequencing

A total of 2 million of PDAC cells (BxPC‐3 and CFPAC‐1) transfected with circFOXP1‐specific siRNAs or control siRNAs were collected using Trizol Reagents. After isolating total RNAs, RNA concentration and purity were measured using NanoDrop 2000 (Thermo Fisher Scientific, Wilmington, DE) and RNA integrity was assessed using the RNA Nano 6000 Assay Kit of the Agilent Bioanalyzer 2100 system (Agilent Technologies, CA, USA). A total amount of 1 μg RNA per sample was used for generating sequencing libraries using NEBNext UltraTM RNA Library Prep Kit for Illumina (NEB, USA) following the manufacturer's recommendations and index codes were added to attribute sequences to each sample. The clustering of the index‐coded samples was performed on a cBot Cluster Generation System using TruSeq PE Cluster Kit v4‐cBot‐HS (Illumia) according to the manufacturer's instructions. After cluster generation, the library preparations were sequenced on an Illumina platform and paired‐end reads were generated. The raw reads were further processed with a bioinformatic pipeline tool, BMKCloud (www.biocloud.net) online platform to perform gene functional annotation, differential analysis, and gene enrichment analysis.

### 2.20 Statistical Analysis

The statistical analyses were conducted using the SPSS version 22.0 (IBM Corp., Armonk, NY, USA). Student's *t*‐test or one‐way ANOVA was used to compare group differences on the promise of normal distribution; otherwise, the nonparametric Mann–Whitney U or Kruskal Wallis H test was adopted. Survival analysis was performed using the Kaplan–Meier method, and the log‐rank was carried out to compare the survival curves. The Chi‐squared test was used to analyse the relationship between the circFOXP1 and the clinicopathological parameters of the patients. All data were presented as the mean ± the standard deviation from at least three independent experiments, unless otherwise noted. *p* < 0.05 indicated a statistically significant difference.

## 3 Results

### 3.1 The Identification of circFOXP1 in PDAC

**FIGURE 1:**
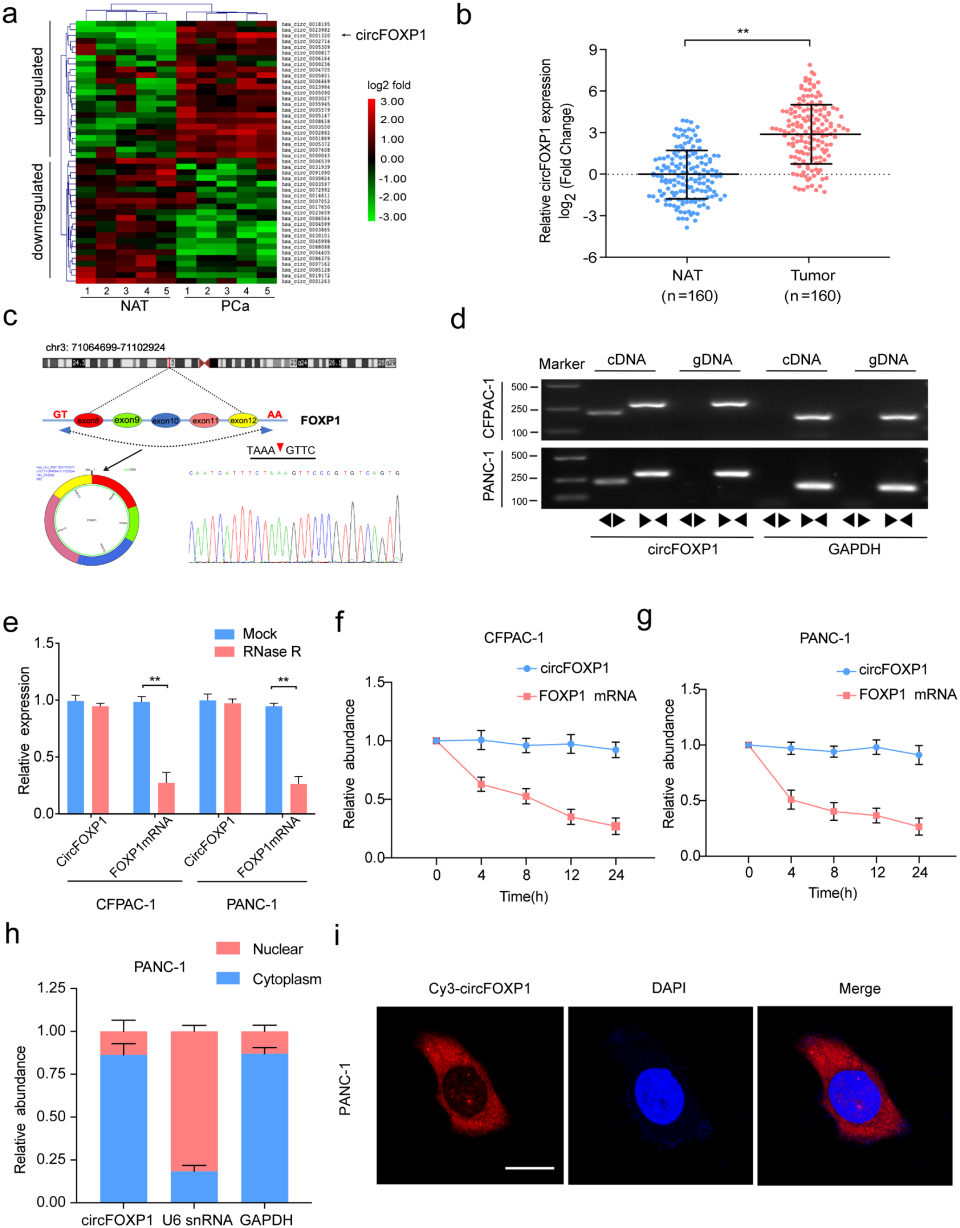
The identification and characterization of circFOXP1 in PDAC. (a) A heatmap showing differentially expressed circRNAs in 5 pairs of PDAC tissues and NATs. The red and green scales represent higher or lower expression levels, respectively. (b) qRT‐PCR analysis of circFOXP1 expression in 160 paired PDAC and NATs. The data are presented in the logarithmic form of relative expression. (c) Schematic illustration showing the genomic loci of the FOXP1 gene and the circFOXP1 derived from exon 8 to 12 of FOXP1. A red arrow indicates the back‐splicing site of circFOXP1, which was validated by Sanger sequencing. (d) Complementary DNA (cDNA) or genomic DNA (gDNA) that were isolated from CFPAC‐1 and PANC‐1 cells were applied for PCR amplification using divergent and convergent primers, followed by electrophoresis assays that corroborated the presence of circFOXP1. (e) qRT‐PCR analysis showing higher resistance of circFOXP1 to RNase R compared with linear FOXP1. (f, g) qRT‐PCR analysis to assess the stability of circFOXP1 and FOXP1 mRNA in CFPAC‐1 and PANC‐1 cells after Actinomycin D treatment for indicated duration. (h) The cellular distribution of circFOXP1 was examined using a subcellular fractionation assay and the results of RT‐PCR analysis indicated that circFOXP1 is mainly located in the cytoplasm. (with GAPDH as a cytoplasmic control and U6 snRNA as a nuclear control). (i) Representative FISH images showing the subcellular localization of circFOXP1. The circFOXP1 probe was labelled with Cy3 dye (red), and nuclei were stained with DAPI (blue). The images were photographed at 1000× magnification. Scale bar = 10 μm. The error bars represent the standard deviations of three independent experiments. *p < 0.05, **p < 0.01.

To search for critical circRNAs in PDAC, five pairs of PDAC tissues and normal adjacent tissues (NATs) were subjected to a circRNA microarray. With an absolute value of log_2_ fold change ≥ 1 and an adjusted *p* value < 0.05 as the cut‐off threshold, we identified a total of 46 differentially expressed circRNAs (Figure 1a). The top 15 candidate circRNAs were selected for further validation because their parental genes are also highly expressed in PDAC in the TCGA database and predict a dismal prognosis for PDAC patients. Subsequently, hsa_circ_0018195, hsa_circ_0023982, and hsa_circ_0001320 were selected for further validation in a cohort of 160 PDAC patients, and qRT‐PCR analysis revealed that only hsa_circ_0001320 (termed circFOXP1) was abnormally upregulated in PDAC tissue samples in comparison with NATs (Figure 1b).

circFOXP1 was formed by the back‐splicing of exon 8 to exon 12 of the Forkhead Box P1 (FOXP1) gene with a length of 692 nt, and the back‐splicing junction site of circFOXP1 was confirmed by Sanger sequencing (Figure 1c). Nucleic acid electrophoresis showed that circFOXP1 could only be amplified by divergent primers from complementary DNA (cDNA) but not from genomic DNA (gDNA) (Figure 1d). As shown in Figure 1e, circFOXP1 was more resistant to RNase R treatment than its linear form. To further verify the stability of circFOXP1, actinomycin D was used to suppress RNA transcription. And the results showed that, compared with linear FOXP1, circFOXP1 appeared to be greatly stable in PDAC cells (Figure 1f,g). In addition, the cellular localization of circFOXP1 was investigated by subcellular fractionation and FISH assays, which revealed the enrichment of circFOXP1 in the cytoplasm of PDAC cells (Figure 1h,i). Altogether, these results demonstrated that cytoplasm‐distributed circFOXP1 is a highly upregulated circRNA in PDAC.

### 3.2 circFOXP1 Fuels the Proliferation, Migration and Invasion of PDAC Cells In Vitro

**FIGURE 2:**
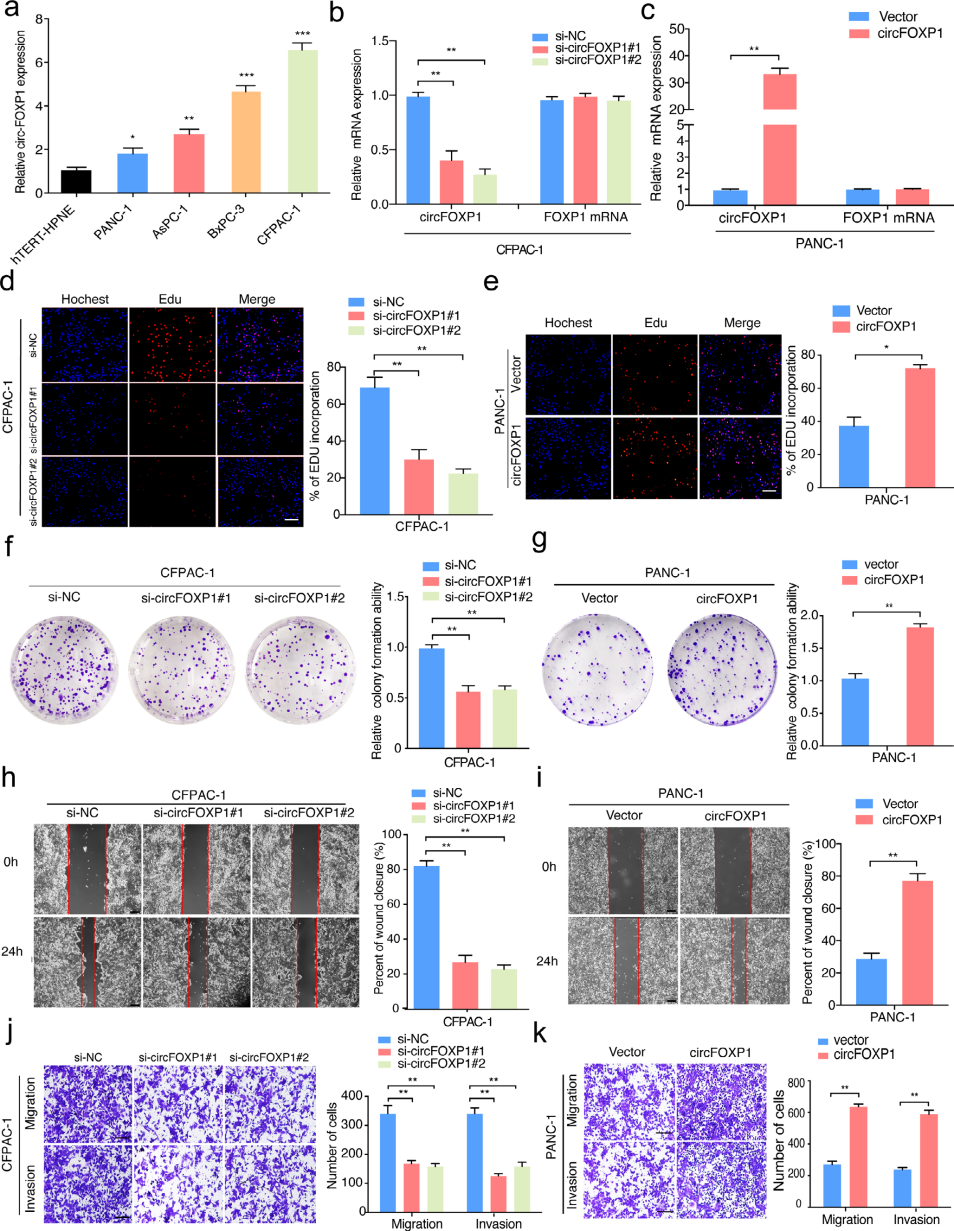
circFOXP1 promotes proliferation, migration and invasion of PDAC cells in vitro. (a) qRT‐PCR analysis for the expression of circFOXP1 in pancreatic epithelial cells (hTERT‐HPNE) and PDAC cell lines (PANC‐1, AsPC‐1, BxPC‐3 and CFPAC‐1). (b) The expression of circFOXP1 and FOXP1 mRNA was assessed by qRT‐PCR in CFPAC‐1 cells transfected with scramble or circFOXP1‐specific siRNAs. (c) qRT‐PCR analysis for the expression of circFOXP1 and FOXP1 mRNA in PANC‐1 cells transfected with the control or circFOXP1 plasmid. (d) EdU assays showing that knockdown of circFOXP1 inhibited the proliferation of CFPAC‐1 cells. (e) Representative images of EdU incorporation assays indicating that overexpression of circFOXP1 promoted DNA synthesis in PANC‐1 cells. Scale bar = 100 μm. (f, g) Colony formation assays to evaluate the cell proliferation ability after knocking down circFOXP1 in CFPAC‐1 cells (f) and overexpressing circFOXP1 in PANC‐1 cells (g). (h, i) Representative images of wound healing assays showing that the migration capability of CFPAC‐1 cells was suppressed after being treated with circFOXP1‐specific siRNAs (h). By contrast, PANC‐1 cells transfected with the circFOXP1 plasmid exhibited an increased migration rate (i). The images were photographed at 40× magnification. Scale bar = 200 μm. (j, k) The migration and invasion ability of PDAC cells were assessed using the Transwell migration and invasion assays after knocking down circFOXP1 in CFPAC‐1 cells (j) and overexpressing circFOXP1 in PANC‐1 cells (k). The images were photographed at 100× magnification. Scale bar = 100 μm. Statistical significance was assessed using two‐tailed *t*‐tests for two group comparison, and one‐way ANOVA, followed by Dunnett's tests for multiple comparison. The error bars represent the standard deviations of three independent experiments. *p < 0.05, **p < 0.01.

To investigate whether circFOXP1 affects the malignant behaviours of PDAC cells in vitro, we first evaluated the abundance of circFOXP1 in a set of PDAC cell lines and a normal pancreatic ductal cell line, hTERT‐HPNE. qRT‐PCR analysis showed that circFOXP1 was upregulated in PDAC cells (PANC‐1, AsPC‐1, BxPC‐3 and CFPAC‐1) compared with the normal pancreatic cell line (Figure 2a). We then specifically downregulated circFOXP1 expression in CFPAC‐1 and BxPC‐3 cells (Figure 2b, Figure S1a) with two small interfering RNAs targeting the back‐splicing site of circFOXP1 or overexpressed circFOXP1 in PANC‐1 cells using a circFOXP1 plasmid (Figure 2c) without altering FOXP1 expression. EdU incorporation assay indicated that circFOXP1 downregulation inhibited the proliferation of PDAC cells (Figure 2d, Figure S1b), while ectopic expression of circFOXP1 showed the opposite effect on PDAC cells (Figure 2e). Colony formation assays showed similar results (Figure 2f,g). Next, we assessed whether circFOXP1 could influence the PDAC cells' migration and invasion abilities. We found that knockdown of circFOXP1 impaired the migration of PDAC cells by using wound healing assays (Figure 2h), which was in line with the results of Transwell assays (Figure 2j, Figure S1c). By contrast, circFOXP1 overexpression led to opposite results (Figure 2i,k). Collectively, these results suggest that circFOXP1 promotes the proliferation, migration and invasion of PDAC cells in vitro.

### 3.3 circFOXP1 Accelerates Tumour Growth and Metastasis of PDAC In Vivo

**FIGURE 3:**
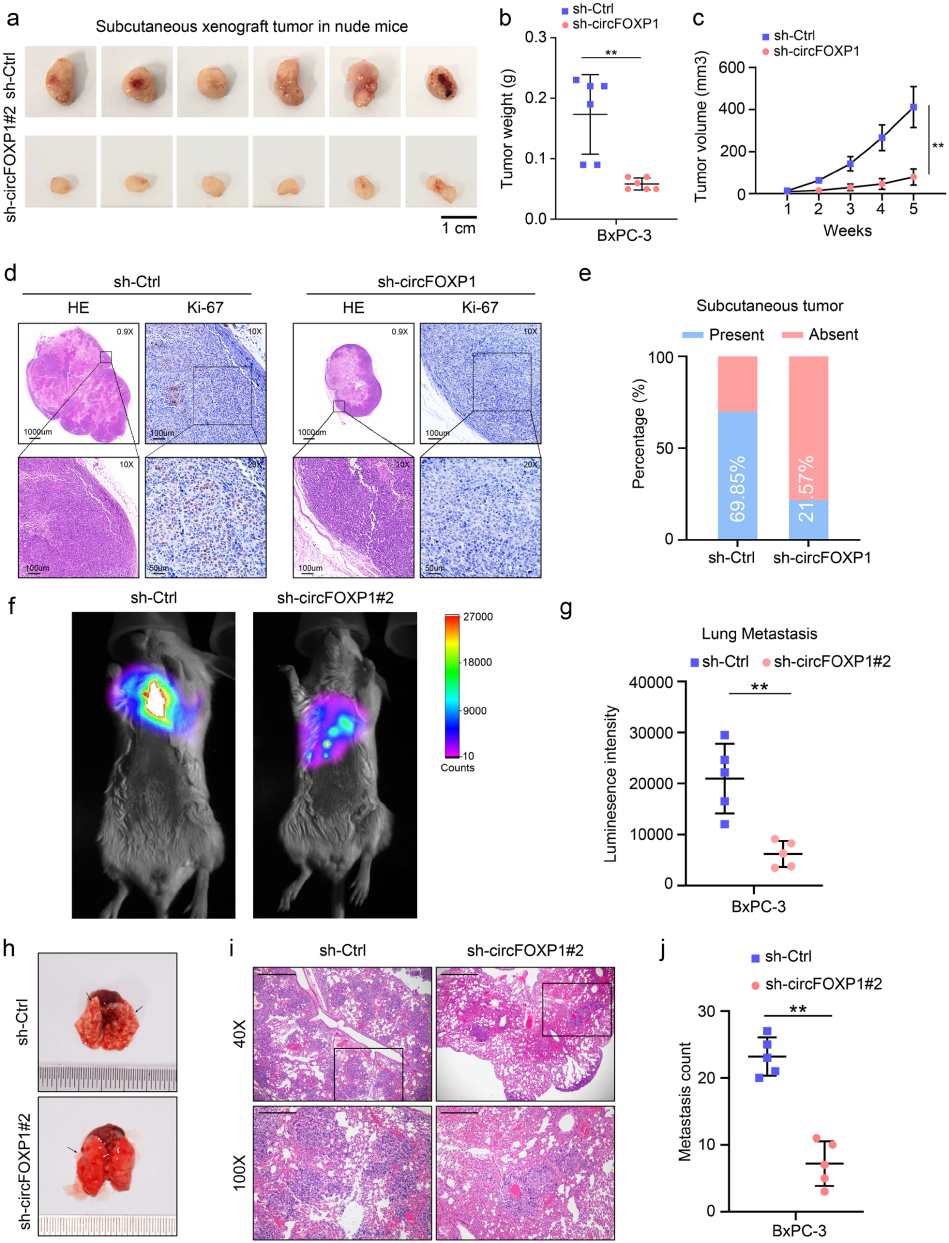
circFOXP1 accelerates tumour growth and metastasis of PDAC in vivo. (a) Subcutaneous xenograft tumours in both groups were exercised and photographed. (b, c) The tumour weight and volume decreased dramatically in the sh‐circFOXP1 group compared with the control group. (d, e) Representative HE and IHC staining images of subcutaneous tumours revealed the percentage of Ki‐67 positive tumour cells in both groups. The images were photographed at 200× (upper panel) or 400× (lower panel) magnification. (f, g) Representative IVIS images and analysis of luminescence intensity in lung in the tail‐vein injection model (*n* = 5 for each group). (h) Representative macroscopic images of lung metastatic tumours, as indicated by black arrows. (i) HE staining of lung metastatic tumours. The images were photographed at 40× (upper panel) or 100× (lower panel) magnification. (j) The number of lung metastatic tumours decreased significantly in the sh‐circFOXP1 group. **p < 0.01.

To evaluate the tumourigenic function of circFOXP1 in vivo, we subcutaneously injected BxPC‐3 cells carrying circFOXP1‐targeting shRNAs or control shRNA into the left hind flank of BALB/c nude mice to establish a xenograft mouse model. The results showed that knockdown of circFOXP1 reduced tumour growth (Figure 3a), as indicated by a decrease in tumour weight (Figure 3b) and growth curve (Figure 3c). IHC staining showed that circFOXP1 knockdown led to a decreased number of tumour cells positive for Ki‐67 (Figure 3d,e).

We next investigated the impact of circFOXP1 on the metastasis of PDAC cells in vivo by employing a tail vein injection model. As shown in Figure 3f,g, inhibiting circFOXP1 impeded the dissemination of PDAC cells in vivo. Mice in the circFOXP1 knockdown group showed weaker luminescent signals compared to the control group. Consistently, microscopic examination revealed a lower number of metastatic foci in the sh‐circFOXP1 group compared to the control group (Figure 3h–j). Taken together, circFOXP1 promotes the tumour formation and metastasis of PDAC in vivo.

### 3.4 circFOXP1 Sponges miR‐320b in PDAC Cells

**FIGURE 4:**
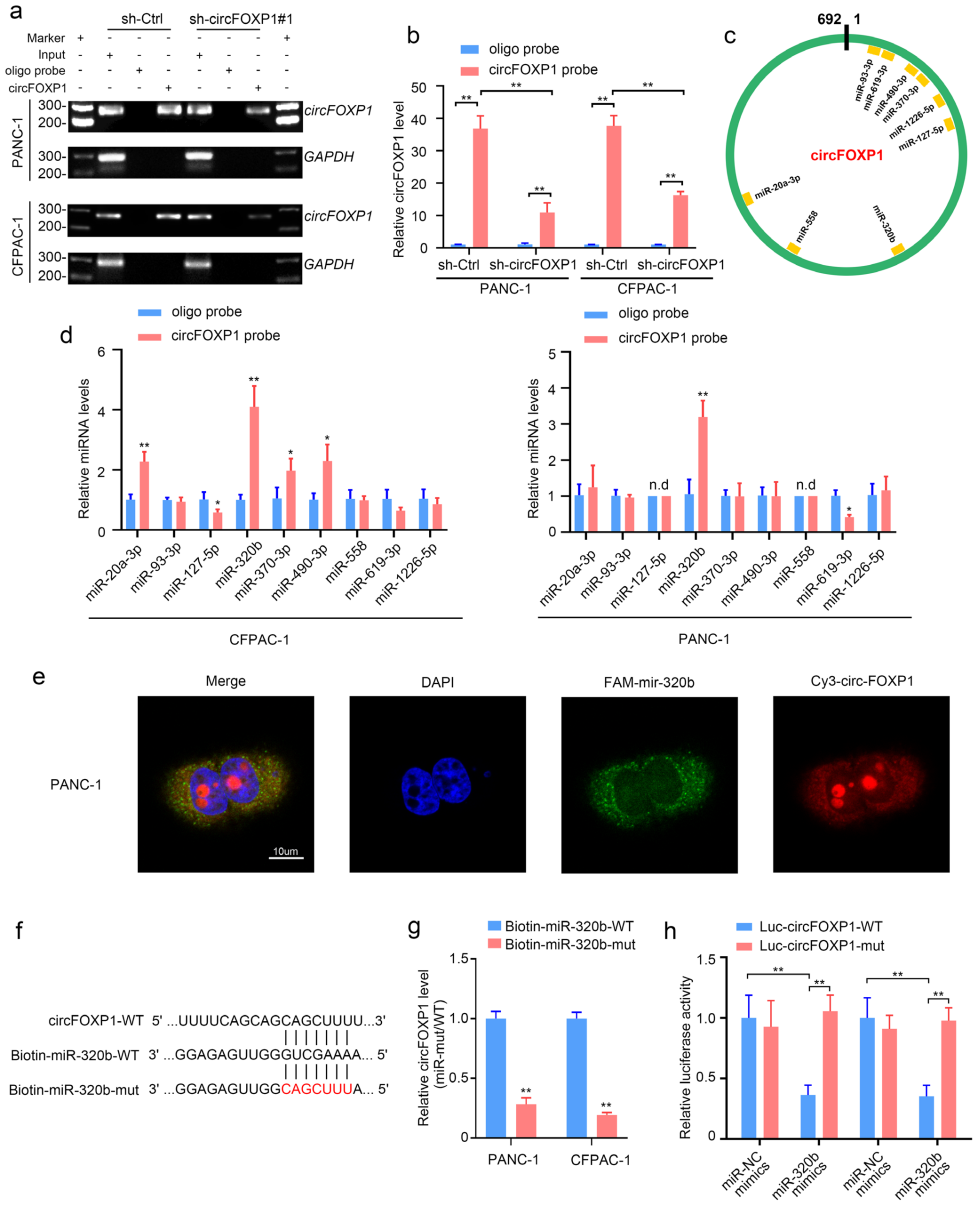
circFOXP1 serves as a sponge for miR‐320b in PDAC cells. (a, b) The specificity and efficiency of the circFOXP1 probe was confirmed using gel electrophoresis assay and qRT‐PCR in PANC‐1 and CFPAC‐1 cells. (c) Schematic illustration showing potential target miRNAs of circFOXP1 predicted by database tools including miRanda, starbase and RNA hybrid. (d) The circFOXP1‐interacting miRNAs were pulldown using circFOXP1 probes and the eluted RNA complex was extracted, reversely transcribed followed by qRT‐PCR analysis to identify the enrichment of nine potential target miRNAs in CFPAC‐1 and PANC‐1 cells. (e) The co‐localization of circFOXP1 and miR‐320b in PANC‐1 cells was detected using a FISH assay. circFOXP1 probes were labelled with Cy3 and miR‐320b probes were labelled with FAM. Nuclei were stained with DAPI. (f) Schematic illustration showing the predicted binding site of circFOXP1 and miR‐320b. The bases in red indicated the mutated sequence that may abolish the interaction between circFOXP1 and miR‐320b. (g) Biotinylated miRNA pull‐down (WT or mut) and qRT‐PCR assays showing the enrichment levels of circFOXP1 after co‐transfection of circFOXP1 and miR‐320b mimics in PANC‐1 and CFPAC‐1 cells. GAPDH was used as the negative control. (h) The luciferase activities of the circFOXP1 luciferase reporter vector (WT or mut) were measured after transfection with miR‐320b mimics or NC mimics into PANC‐1 and CFPAC‐1 cells. *p < 0.05, **p < 0.01.

Previous studies have shown that circRNAs primarily exert their functions in tumorigenesis by sponging miRNAs associated with cancers [16, 17]. To identify miRNAs that interact with circFOXP1, we use a biotin‐labelled circFOXP1 probe and a negative probe in PDAC cells to conduct RNA pull down assays. The results showed the exclusive binding of circFOXP1 probe to its transcripts, with diminished capture in circFOXP1‐depleted cells, thus confirming the specificity and efficacy of circFOXP1 probe (Figure 4a,b). We then predicted possible circFOXP1‐interacting miRNAs by searching three public databases, yielding nine candidate miRNAs selected for further validation (Figure 4c). We identified that miR‐320b was the only miRNA that bound to circFOXP1 in both CFPAC‐1 and PANC‐1 cells through RNA pulldown assays followed by qRT‐PCR (Figure 4d). FISH assays further demonstrated the co‐localization of circFOXP1 and miR‐320b in the cytoplasm of PDAC cells (Figure 4e). The reciprocal interaction between circFOXP1 and miR‐320b was validated by miRNA pulldown assay with biotinylated wild‐type (WT) and mutant (mut) miR‐320b (Figure 4f,g), and by a luciferase reporter assay using WT or a mutant reporter (Figure 4h). Collectively, these data showed that circFOXP1 serves as a miR‐320b sponge in PDAC.

### 3.5 miR‐320b Suppresses Proliferation, Migration and Invasion of PDAC Cells In Vitro

**FIGURE 5:**
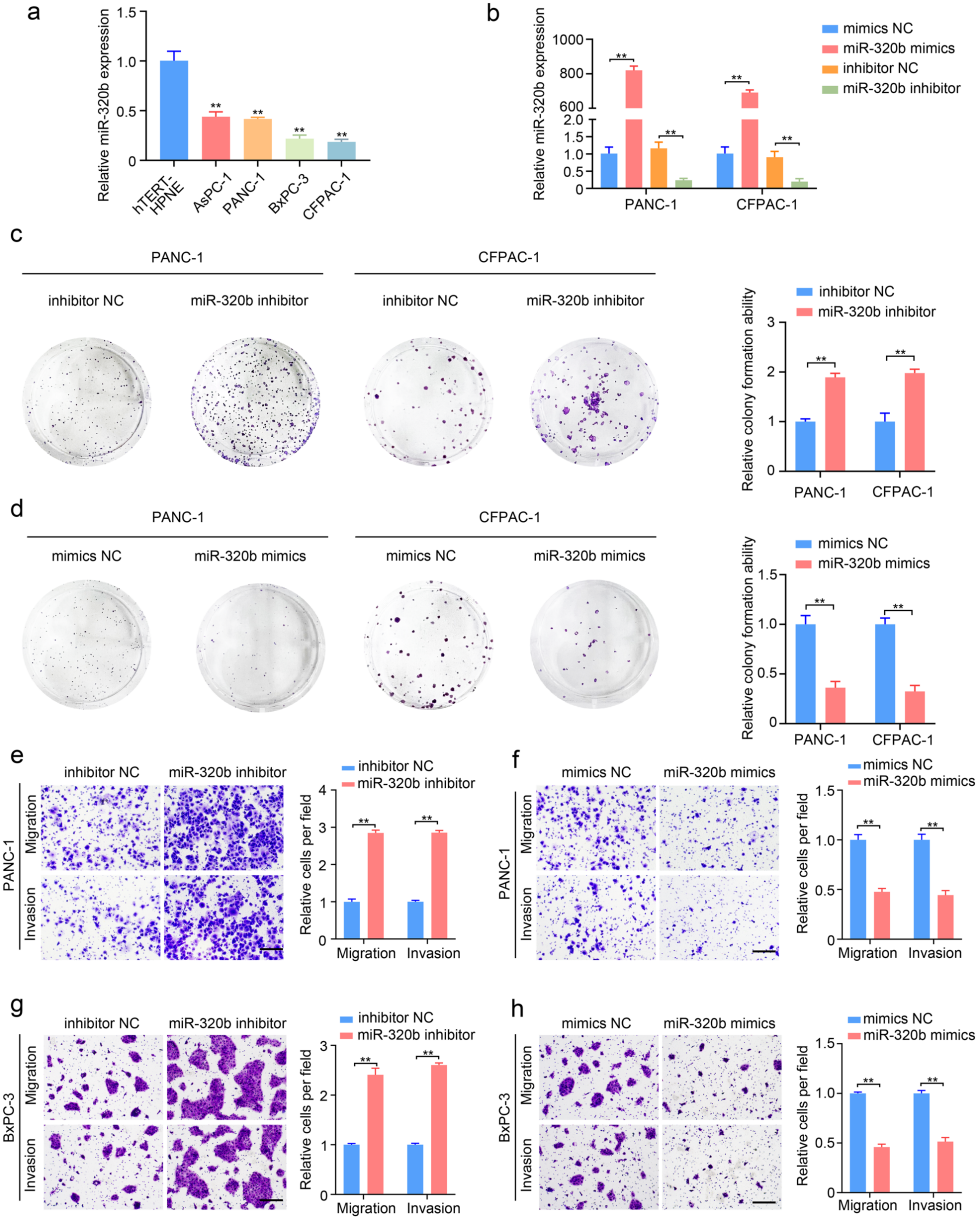
miR‐320b suppresses the proliferation, migration, and invasion of PDAC cells in vitro. (a) qRT‐PCR analysis of the relative expression levels of miR‐320b in pancreatic epithelial cells (hTERT‐HPNE), PDAC cells (Aspc‐1, PANC‐1, BxPC‐3 and CFPAC‐1). (b) qRT‐PCR analysis of the expression levels of miR‐320b in PANC‐1 and CFPAC‐1 cells transfected with miR‐320b mimics or inhibitors. PDAC cells transfected with scramble mimics or inhibitors were used as the negative control. (c, d) The colony formation ability of PDAC cells was evaluated using colony formation assays after transfection with miR‐320b mimics or inhibitors in PANC‐1 and CFPAC‐1 cells. (e–h) Representative images of Transwell migration and invasion assays showing the effect of migratory and invasive of PANC‐1 and CFPAC‐1 cells transfected with miR‐320b mimics or inhibitors. Scale bar = 100 μm. **p < 0.01.

miR‐320b has been shown to suppress tumour progression in various cancers [18, 19, 20]. To investigate its role in PDAC, we first examined the expression and biological functions of miR‐320b in PDAC. qRT‐PCR analysis showed significantly lower levels of miR‐320b in PDAC cells compared to normal pancreatic ductal cells (Figure 5a). We then validated the efficacy of miR‐320b mimics and inhibitor (Figure 5b). Colony formation assays indicated that miR‐320b knockdown promoted the proliferation of PDAC cells (Figure 5c), while miR‐320b mimics showed the opposite effect (Figure 5d). Furthermore, miR‐320b inhibitor‐transfected PDAC cells exhibited enhanced migration and invasion abilities (Figure 5e–g). Conversely, miR‐320b mimic transfection led to impaired migration and invasion in PDAC cells (Figure 5f–h). These data collectively demonstrate that miR‐320b acts as a tumour suppressor in PDAC cells.

### 3.6 circFOXP1 Upregulates EREG Expression to Activate the MAPK Pathway in PDAC

**FIGURE 6:**
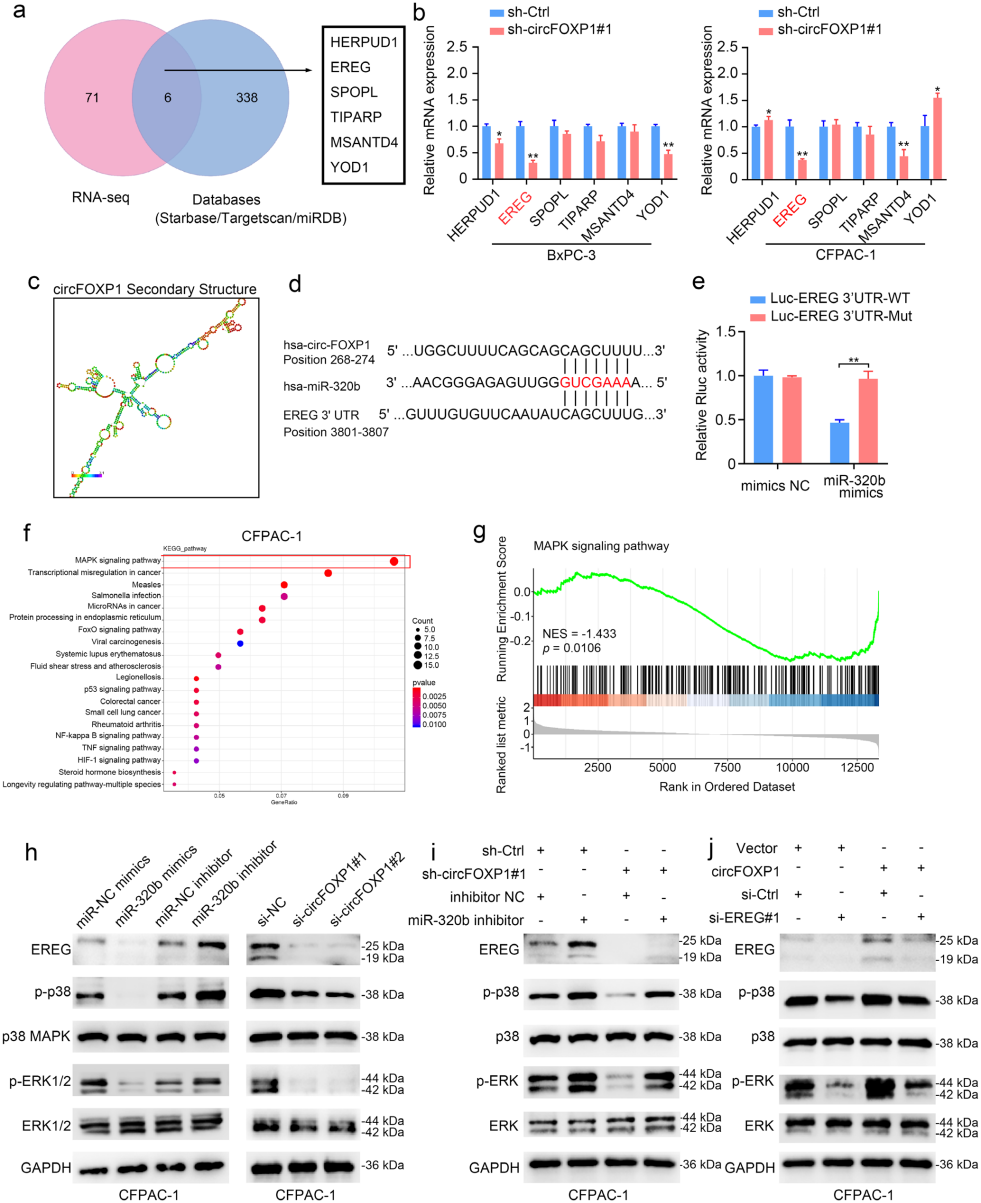
circFOXP1 upregulates EREG expression to activate the MAPK pathway in PDAC. (a) Venny diagram of six potential target genes of miR‐320b identified by overlapping differentially expressed genes from our RNA‐seq data with predicted candidates from Starbase, Targetscan and mirDB databases. (b) qRT‐PCR analysis of the expression changes in above predicted target genes in BxPC‐3 and CFPAC‐1 cells stably transfected with sh‐circFOXP1 and control shRNA. (c) Proposed secondary structure of circFOXP1. (d) Schematic illustration of the predicted binding site of miR‐320b within EREG mRNA and circFOXP1. Red bases indicate the potential mutated sequence that may abolish the interaction between miR‐320b and EREG mRNA. (e) Luciferase reporter assay showing the activities of the EREG mRNA 3′ UTR luciferase reporter vector (WT or mut) following transfection with miR‐320b mimics or negative control mimics in PANC‐1 cells. (f) KEGG enrichment analysis of differentially expressed genes in CFPAC‐1 cells after circFOXP1 knockdown. (g) Gene set enrichment analysis (GSEA) showing the enrichment of the MAPK signalling pathway in CFPAC‐1 cells treated with sh‐circFOXP1. (h) Western blotting analysis of EREG, p‐p38, p38, ERK1/2 and p‐ERK1/2 level in CFPAC‐1 cells following transfection with miR‐320b mimics, inhibitors, or circFOXP1‐specific siRNAs. GAPDH served as the internal control. (i) Western blotting analysis of EREG, p‐p38, p38, ERK1/2 and p‐ERK1/2 levels in CFPAC‐1 cells following with circFOXP1‐specific shRNA, miR‐320b inhibitors, circFOXP1 plasmid or siRNAs targeting EREG. (j) The upregulation of EREG and p‐p38, p‐ERK1/2 in CFPANC‐1 cell transfected with circFOXP1 was reversed by silencing EREG, as detected using western blotting. GAPDH was used as the internal control. *p < 0.05, **p < 0.01.

To further explore the downstream target of the circFOXP1/miR‐320b axis, RNA‐sequencing analysis was performed in BxPC‐3 and CFPAC‐1 PDAC cell lines transfected with circFOXP1‐specific siRNAs or control oligonucleotides. Differentially expressed genes identified in both cell lines were intersected with predicted miR‐320b targets using public databases (Starbase, TargetScan and miRDB), revealing 6 candidate genes (Figure 6a). qRT‐PCR analysis further confirmed that EREG was the only gene downregulated in both cell lines following circFOXP1 knockdown (Figure 6b). Notably, EREG was overexpressed in PDAC tissues (Figure S2a) and correlated with worse survival for patients with PDAC (Figure S2b). We selected EREG for further investigation on the basis of these findings.

miRNAs are known to exert regulatory functions by binding to the 3′‐end untranslated region (3′ UTR) of target genes [21, 22, 23]. Thus, analysis of the sequence of circFOXP1, miR‐320b and EREG 3′ UTR region was first conducted. Within circFOXP1 and the 3′ UTR region of EREG, we found an identical sequence CAGCUUU which was complementary to miR‐320b sequence (Figure 6c,d), suggesting that EREG was possibly a common downstream target of both miR‐320b and circFOXP1. To investigate whether miR‐320b directly binds to the EREG 3′ UTR and regulates its expression, we conducted luciferase reporter assays using wild‐type or mutant EREG 3′ UTR luciferase reporter plasmids. Co‐transfection of miR‐320b mimics with wild‐type EREG plasmid decreased luciferase activity remarkably. In contrast, cells transfected with mutant EREG plasmid were resistant to miR‐320b induction and exhibited luciferase activity similar to control groups (Figure 6e). These results suggested that miR‐320b binds to the predicted site of EREG 3′ UTR, thereby confirming EREG as a direct downstream target of miR‐320b.

EREG is documented to bind to EGFR to activate downstream signalling pathways, including PI3K/AKT, MEK/ERK, and STAT signalling pathways [24, 25, 26]. We next investigate the potential role of circFOXP1 in regulating EREG expression and downstream pathways. We use RNA‐seq to analyse Kyoto Encyclopedia of Genes and Genomes (KEGG) enrichment, which indicated that the MAPK signalling pathways were significantly enriched in both BxPC‐3 and CFPAC‐1 cells (Figure 6f, Figure S2c). Gene set enrichment analysis (GSEA) further confirmed that the MAPK signalling pathways were significantly downregulated in circFOXP1 knockdown PDAC cells (Figure 6g, Figure S2d). Meanwhile, western blotting further showed that EREG knockdown markedly reduced MAPK levels, as well as the phosphorylated levels of its downstream signalling transducers, p38 and ERK1/2, without affecting the levels of total p38 and ERK1/2 (Figure S2e). Consistently, western blotting analysis demonstrated that both miR‐320b overexpression and circFOXP1 knockdown remarkedly decreased EREG protein abundance and inhibited activation of the MAPK signalling pathways, as evidenced by reduced phosphorylation of p38 and ERK1/2. Conversely, PDAC cells transfected with miR‐320b inhibitors exhibited elevated EREG protein levels and enhanced phosphorylation of p38 and ERK1/2 in CFPAC‐1 and BxPC‐3 cells (Figure 6h, Figure S2f). Importantly, circFOXP1 silencing‐induced inactivation of MAPK signalling was partially alleviated by miR‐320b knockdown, while miR‐320b overexpression suppressed MAPK signalling activation induced by ectopic circFOXP1 expression (Figure 6i). Specifically, CFPAC‐1 cells overexpressing circFOXP1 showed increased phosphorylation of p38 and ERK1/2, which was reduced when EREG was knocked down (Figure 6j). Taken together, these data indicated that circFOXP1 antagonizes miR‐320b to upregulate EREG expression and activate the downstream MAPK signalling pathway in PDAC.

### 3.7 circFOXP1 Sustains Malignant Phenotypes of PDAC via Modulating the miR‐320b/EREG Axis

**FIGURE 7:**
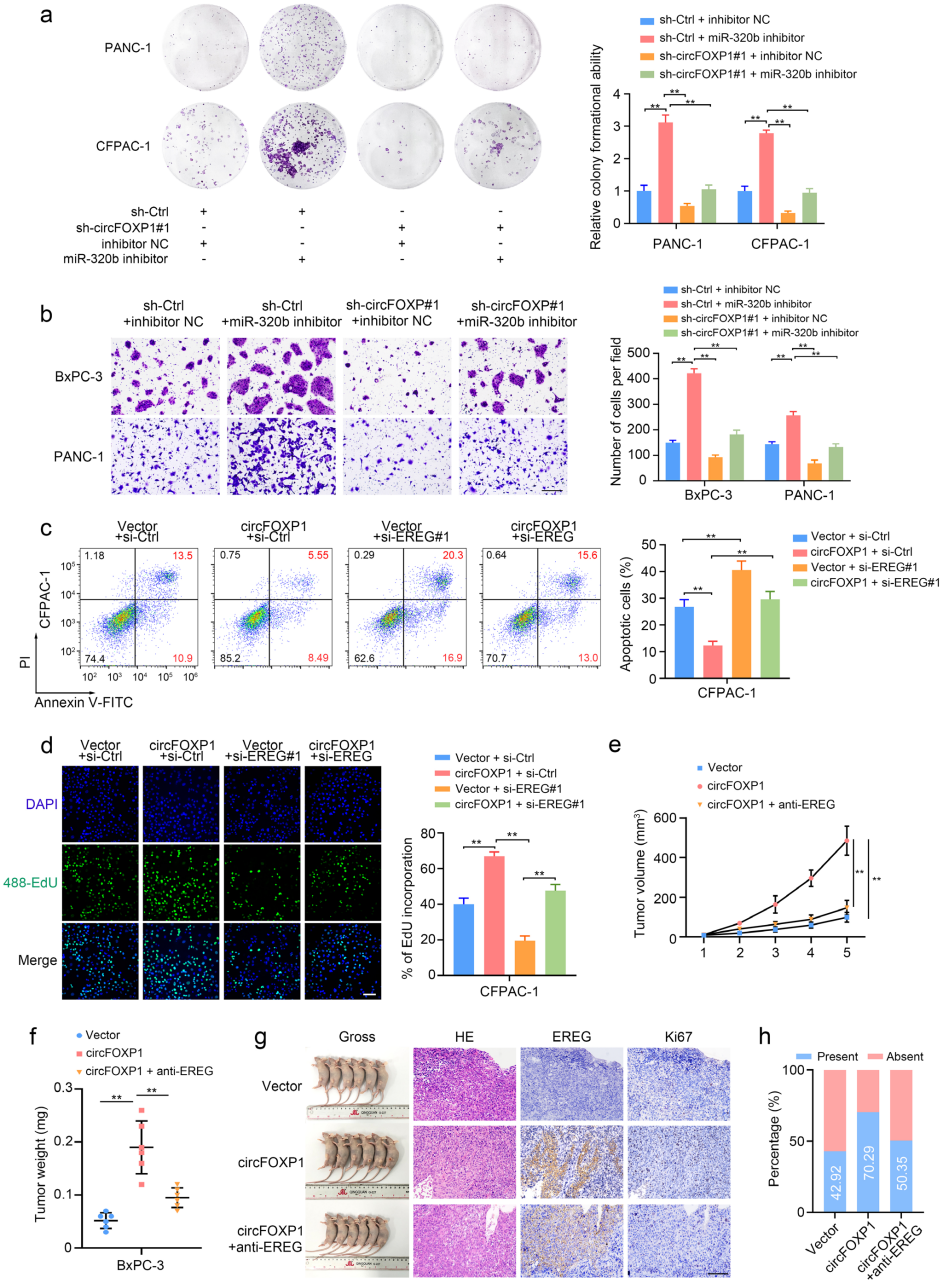
circFOXP1 sustains malignant phenotypes of PDAC via modulating the miR‐320b/EREG axis. (a) Representative image of colony formation assays showing that circFOXP1 knockdown inhibited the colony formation ability of PANC‐1 and CFPAC‐1 cells; however, this effect was reversed after co‐transfection with miR‐320b inhibitors. (b) The migratory and invasive ability of BxPC‐3 and PANC‐1 cells was assessed using the Transwell migration and invasion assays that demonstrated the suppressive effect of circFOXP1 knockdown on PDAC cells, which was reversed after co‐transfection with miR‐320b inhibitors. (c) The impact of circFOXP1 overexpression and EREG knockdown on the percentage of apoptotic CFPAC‐1 cells was evaluated by Flow cytometry. (d) Representative image of Edu incorporation assays showing that circFOXP1 overexpression promoted the proliferation of CFPAC‐1 cells; however, this effect was reversed after EREG knockdown. (e, f) Compared with the control group, the tumour weight and volume increased significantly in the circFOXP1 overexpression group, which was reversed by the treatment of EREG neutralizing antibody. (g) Representative images of subcutaneous xenograft tumours and HE and IHC staining images of subcutaneous tumours revealing the percentage of Ki‐67 positive tumour cells in different groups. (h) Percentage bar chart showing the percentage of Ki‐67 positive tumour cells in different groups. *p < 0.05, **p < 0.01.

To determine whether circFOXP1‐mediated miR‐320b sequestration contributes to PDAC progression, we examined the malignant phenotypes in circFOXP1 knockdown PDAC cells. Colony formation assays revealed that the colony‐forming ability of PDAC cells was suppressed through circFOXP1 knockdown, while this effect was abolished after transfecting with miR‐320b inhibitors (Figure 7a). Similarly, the impaired migration and invasion of PDAC cells induced by circFOXP1 interference were reversed by the downregulation of miR‐320b (Figure 7b). These results suggested that circFOXP1 promotes the proliferation, migration, and invasion of PDAC cells by antagonizing the tumour‐suppressing effects of miR‐320b.

Since our above results have established that EREG is a downstream target of the circFOXP1/miR‐320b axis, we further confirmed whether the EREG pathway is essential for circFOXP1‐mediated PDAC progression. To this end, PDAC cells with or without stable circFOXP1 overexpression were transfected with specific siRNAs targeting EREG or control siRNAs. Notably, circFOXP1 overexpression‐induced activation of the MAPK signalling was partially abrogated by EREG downregulation (Figure S2g). Further experiments revealed that circFOXP1 overexpression protected PDAC cells from apoptosis and promoted PDAC cell proliferation (Figure 7c,d). Importantly, these effects were significantly reversed by EREG knockdown. To investigate whether EREG was necessary for the in vivo tumour‐promoting role of circFOXP1 in PDAC, we blocked the EREG signalling using a neutralizing antibody against EREG. Ectopic expression of circFOXP1 accelerated the PDAC cells' growth in vivo, as evidenced by increased tumour weight and volume (Figure 7e,f). The administration of EREG‐neutralizing antibody significantly abolished tumour progression fueled by overexpression of circFOXP1. Moreover, IHC staining indicated that circFOXP1 overexpression significantly increased the protein levels of EREG and Ki‐67 in PDAC cells, and these effects were significantly reversed by anti‐EREG treatment (Figure 7g,h). Collectively, our data suggested that circFOXP1 exerts its biological functions by the regulation of the miR‐320b/EREG axis.

### 3.8 circFOXP1 Overexpression Correlates With Poor Prognosis of PDAC Patients

**FIGURE 8:**
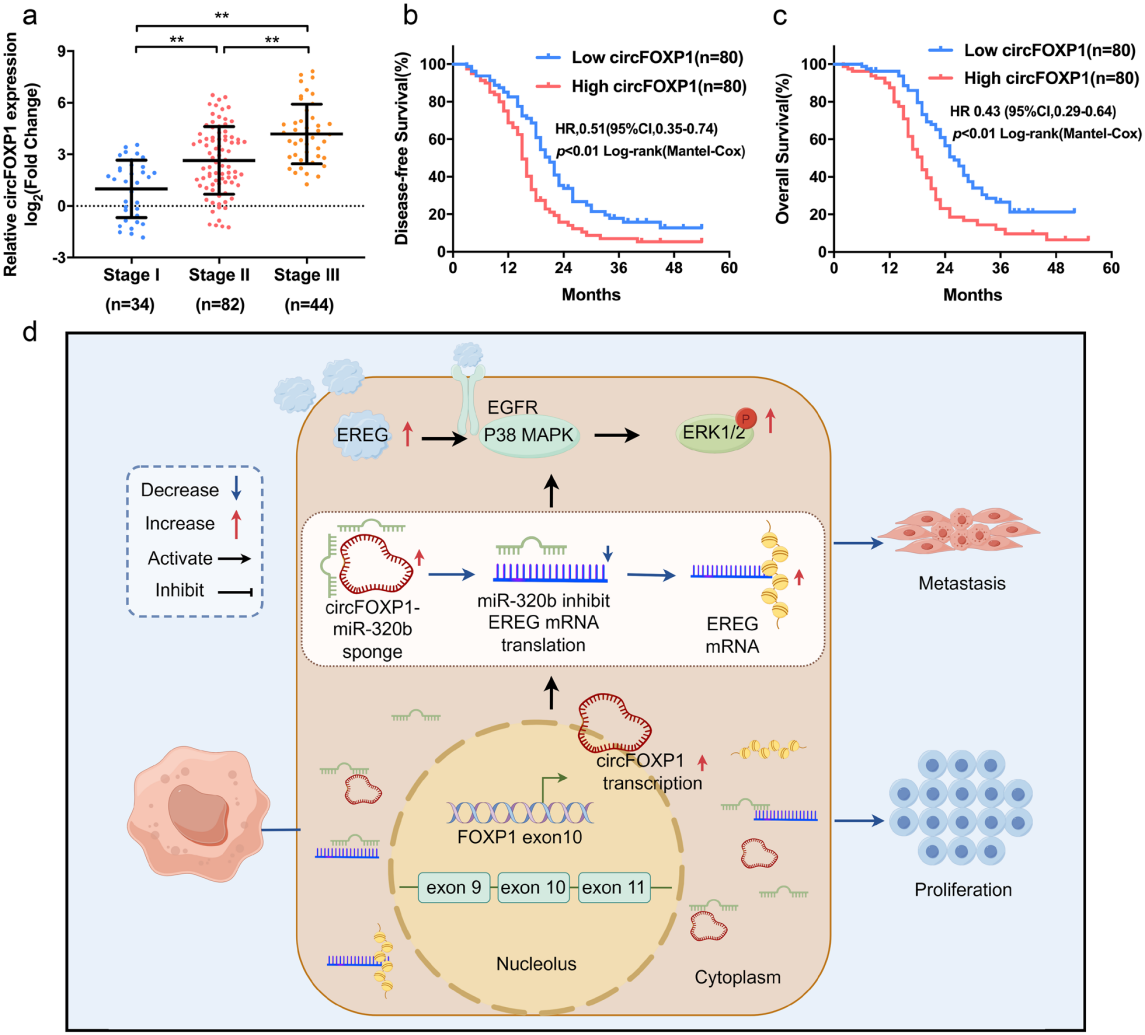
CircFOXP1 overexpression correlates with poor prognosis of PDAC patients. (a) qRT‐PCR analysis of circFOXP1 expression in PDAC tissues diagnosed at different TNM stages. (b, c) Kaplan–Meier curves for OS (a) and DFS (b) of patients with PDAC with low versus high circFOXP1 expression. The median circFOXP1 expression was used as the cut‐off value. Nonparametric Mann–Whitney U‐test was employed for statistical analysis. (d) Schematic illustration showing the mechanism by which circFOXP1 promotes proliferation and metastasis in PDAC via the miR‐320b/EREG axis.

To further evaluate the clinical relevance of circFOXP1 in our PDAC cohort, we first correlated its expression with clinicopathological characteristics of patients with PDAC (Table S6). circFOXP1 expression was found to be positively associated with TNM stage (Figure 8a).

Importantly, Kaplan–Meier analysis demonstrated that higher circFOXP1 expression predicted dismal overall survival (OS) and disease‐free survival (DFS) for patients with PDAC (Figure 8b,c). These clinical data suggested that circFOXP1 could serve as a potential biomarker for predicting the prognosis of PDAC patients (Figure 8d).

## 4 Discussion

CircRNA have emerged as important regulators and potential therapeutic targets in PDAC progression. However, our understanding of their specific mechanisms remains incomplete. Here, we identified a novel circRNA, circFOXP1, which is highly expressed in PDAC and correlated with poor prognosis. We found that circFOXP1 serves as a sponge for miR‐320b to upregulate EREG and activate the MAPK/ERK pathway, thereby promoting PDAC cell proliferation and metastasis. Our findings highlight circFOXP1 as a potential biomarker and therapeutic target for PDAC, suggesting promising avenues for developing new treatment strategies.

CircRNAs have emerged as crucial regulators of human diseases, including inflammatory diseases, cardiovascular diseases and various cancers [27, 28, 29]. We also previously identified circBFAR as a critical tumour‐promoting molecule by activating MET (Mesenchymal‐Epithelial Transition factor) pathway [30]. The ability to modulate gene expression, protein abundance and function at multiple levels of cellular activity highlights their significance in pathological conditions. However, the role, mechanism and potential therapeutic target remain unclear for circular RNA as diagnostic and prognostic biomarkers in PDAC. Previous studies have reported the aberrant expression of circFOXP1 in bladder cancer [31], colorectal cancer [32] and osteosarcoma [33], where it has been shown to promote cell proliferation and angiogenesis that facilitate tumour progression. However, the expression and functional role of circFOXP1 in PDAC remain unreported. Here, using high‐throughput RNA sequencing to uncover abnormally expressed circRNAs in patients with PDAC, we identified an uncharacterized circRNA, that is circFOXP1, which is aberrantly overexpressed in PDAC tissues and predicts poor patient prognosis. Our functional experiments identified that circFOXP1 enhances PDAC cell proliferation, migration, invasion and metastasis both in vitro and in vivo. As previously reported, circFOXP1 can regulate protein ubiquitination in promoting the pathological process of brain injury [34], translating the tumour suppressor gene FOXP1 in colon cancer [32], and acting as an RNA‐binding protein (RBP) to stabilize mRNA [35, 36, 37]. Notably, in mesenchymal stem cells, CircFOXP1 can modulate non‐canonical Wnt and EGFR signalling pathways through direct interaction with miR‐17‐3p and miR‐127‐5p [37], emphasizing the sponging mechanism of circFOXP1 in different process. However, the binding and regulatory mechanisms between CircFOXP1 and microRNAs in pancreatic cancer remain unreported. Mechanistically, through our GSEA analysis from RNA‐seq, alongside in vitro and in vivo experiments on two different PDAC cells, we confirmed that circFOXP1 functions as a sponge for miR‐320b, leading to increased EREG expression and activation of the MAPK/ERK pathway, ultimately driving tumour progression. Importantly, inhibiting the EREG pathway abrogated circFOXP1‐mediated aggressive behaviours in PDAC cells. These findings suggest a novel regulatory circFOXP1/miR‐320b/EREG axis, providing a potential therapeutic target for PDAC.

miRNAs are a well‐characterized subfamily of non‐coding RNAs that exert their tumour‐boosting or ‐inhibiting roles by regulating gene expression post‐transcriptionally via binding to target mRNAs untranslated regions (UTR). Emerging evidence suggests that miRNAs serve as diagnostic and prognostic biomarkers in PDAC, potentially for early detection and predicting patient outcomes or responses to chemotherapy [38, 39]. The miR‐320 family, comprising five members (miR‐320a, miR‐320b, miR‐320c, miR‐320d and miR‐320e), is implicated in human malignancies and is considered a tumour suppressor family by modulating cell proliferation, apoptosis, and repressing epithelial‐mesenchymal transition (EMT) [40]. For instance, hsa‐miR‐320a was found to inhibit hepatocellular carcinoma (HCC) progression and metastasis by inactivating the MAPK pathway [41]. Notably, miR‐320b has been shown to influence PDAC cell proliferation, invasion and EMT by targeting Forkhead Box 1 (FOXM1) [42] and ADP ribosylation factor 1 (ARF1) [43]. However, the precise pathological role and underlying mechanism of miR‐320b in PDAC progression remains unclear. In this study, we revealed that miR‐320b is downregulated in PDAC tissues and cell lines. Importantly, we demonstrate that circFOXP1 acts as a sponge for miR‐320b, preventing its suppression of EREG‐induced downstream MAPK/ERK signalling activation. Taken together, our finding highlights a new regulatory mechanism involving the circFOXP1/miR‐320b/EREG/MAPK axis that fueled PDAC aggressiveness.

EGFR, an important cell surface receptor for multiple growth factors, is generally thought to contribute to tumour progression by activating EGFR signalling pathways aberrantly [44]. EREG, an alternative EGFR ligand, is dysregulated in various cancers, including PDAC. It was widely accepted that EREG exerts its function by activating EGFR‐mediated signalling cascades, such as ERK/MAPK, PI3K/AKT and JAK/STAT pathways [45]. EREG was found to be overexpressed in PDAC and stimulate the proliferation [15], migration and invasion [46]. However, the precise mechanisms underlying its upregulation and intricate interactions with other molecules in PDAC progression remain unclear. In the present study, we provide new insights into the role of EREG in PDAC. We demonstrate that miR‐320b directly targets the 3′ UTR of EREG mRNA, as confirmed by RNA pull‐down and dual luciferase reporter assays. This interaction contributes to the observed increased EREG abundance in PDAC. Moreover, we show that EREG plays a crucial role in circFOXP1‐induced PDAC growth and dissemination. Silencing EREG expression significantly inhibited circFOXP1‐mediated proliferation and invasion of PDAC cells. Notably, in vivo studies using EREG‐neutralizing antibodies demonstrated a remarkable reversal of the malignant phenotypes in circFOXP1‐transduced ‐bearing mice. These data show that the circFOXP1/miR‐320b/EREG/MAPK axis drives PDAC progression and metastasis. Our results suggest that circFOXP1 could serve as a promising biomarker for clinical EGFR‐targeting therapy in PDAC.

## 5 Conclusion

Our data indicate that circFOXP1 is a critical regulator in PDAC, which aberrantly activates EREG/MAPK signalling by acting as a molecular sponge for miR‐320b, thus facilitating PDAC progression. The circFOXP1 might be explored as a novel potential biomarker and therapeutic target for the treatment of PDAC.

## Author Contributions

**Quanbo Zhou:** conceptualization, formal analysis, funding acquisition, writing – review and editing, resources, validation. **Qiang Wang:** investigation, funding acquisition, resources. **Fuxu Feng:** methodology, investigation. **Rihua He:** methodology, investigation. **Bingzheng Zhong:** investigation. **Leyi Huang:** methodology, investigation, formal analysis, writing – original draft, validation, visualization, writing – review and editing. **Xiaofeng Guo:** conceptualization, supervision, investigation, data curation, funding acquisition, writing – original draft, writing – review and editing, project administration, resources, validation. **Jingwen Li:** methodology, investigation. **Dan Su:** methodology, investigation, formal analysis, writing – original draft, writing – review and editing, validation, visualization. **Jie Cao:** funding acquisition, resources.

## Funding

This study was supported by grants from the National Natural Science Foundation of China (Grant No. 82203526, 82173236 and 82472893), Natural Science Foundation of Guangdong Province, China (2022A1515220219), Guangzhou High‐level Key Clinical Specialty Construction Project (No. 9), Young Talent Support Project of Guangzhou Association for Science and Technology (QT2024‐036), Science and Technology Projects in Guangzhou (202201010031).

## Ethics Statement

This study was approved by the Medical Ethics Committee of Sun Yat‐sen Memorial Hospital (SYSEC‐KY‐KS‐2021‐172) and Experimental animal ethics committee of Jennio Biotech Co. Ltd. (Approval number: JENNIO‐IACUC‐2022‐A024).

## Consent

Consent was obtained from all authors.

## Conflicts of Interest

The authors declare no conflicts of interest.

## Supporting information


**Figure S1:** CircFOXP1 promotes proliferation, migration and invasion of PDAC cells in vitro. (a) The expression of circFOXP1 and FOXP1 mRNA was assessed by qRT‐PCR in BxPC‐3 cells transfected with scramble or circFOXP1‐specific siRNAs. (b) EdU assays showing that knockdown of circFOXP1 inhibited the proliferation of BxPC‐3 cells. (c) The migration and invasion ability of PDAC cells were assessed using the Transwell migration and invasion assays after knocking down circFOXP1 in BxPC‐3 cells. Scale bar = 100 μm. *p < 0.05, **p < 0.01.
**Figure S2:** CircFOXP1 upregulates EREG expression to activate the MAPK pathway in PDAC. a. KEGG enrichment analysis of differentially expressed genes in CFPAC‐1 cells after circFOXP1 knockdown. b. Representative image of GSEA analysis in BxPC‐3 cells showing the enrichment of the MAPK signalling pathway in BxPC‐3 cells treated with sh‐circFOXP1. (c) The expression level of EREG gene was evaluated using the publicly available database GEPIA2. (d) Using the publicly available database GEPIA2, higher expression of EREG gene was correlated with shorter overall survival and disease‐free survival. (e) Western blotting analysis protein levels of EREG, p‐p38, p38, ERK1/2 and p‐ERK1/2 after transfection with EREG‐specific siRNAs. (f) Western blotting analysis protein levels of EREG, p‐p38, p38, ERK1/2 and p‐ERK1/2 after transfection with miR‐320b mimics, inhibitors or circFOXP1‐specific siRNAs in BxPC‐3 cells. *p < 0.05, **p < 0.01.
**Table S1:** RNA sequences of siRNAs and shRNAs used in this article.
**Table S2:** Sequences of primers used in this article.
**Table S3:** Primary and secondary antibodies used in this article.
**Table S4:** Sequences of FISH probes used in this article.
**Table S5:** Sequences of probes used in RNA‐pulldown experiments.
**Table S6:** Correlation between circFOXP1 expression and clinicopathologic characteristics of PDAC patients.

## Data Availability

The data that support the findings of this study are available from the corresponding author upon reasonable request.
