# Supplementary material for: circFOXP1 Promotes Pancreatic Ductal Adenocarcinoma Progression Through Regulating EREG/MAPK/ERK Axis
Source: J Cell Mol Med. 2026 Jul 6;30(13):e71230. doi: 10.1111/jcmm.71230 (PMC13337542; doi:10.1111/jcmm.71230)
Supplement: Supplementary file 1 — Figure S1: CircFOXP1 promotes proliferation, migration and invasion of PDAC cells in vitro. (a) The expression of circFOXP1 and FOXP1 mRNA was assessed by qRT‐PCR in BxPC‐3 cells transfected with scramble or circFOXP1‐specific siRNAs. (b) EdU assays showing that knockdown of circFOXP1 inhibited the proliferation of BxPC‐3 cells. (c) The migration and invasion ability of PDAC cells were assessed using the Transwell migration and invasion assays after knocking down circFOXP1 in BxPC‐3 cells. Scale bar = 100 μm. *p < 0.05, **p < 0.01. Figure S2: CircFOXP1 upregulates EREG expression to activate the MAPK pathway in PDAC. a. KEGG enrichment analysis of differentially expressed genes in CFPAC‐1 cells after circFOXP1 knockdown. b. Representative image of GSEA analysis in BxPC‐3 cells showing the enrichment of the MAPK signalling pathway in BxPC‐3 cells treated with sh‐circFOXP1. (c) The expression level of EREG gene was evaluated using the publicly available database GEPIA2. (d) Using the publicly available database GEPIA2, higher expression of EREG gene was correlated with shorter overall survival and disease‐free survival. (e) Western blotting analysis protein levels of EREG, p‐p38, p38, ERK1/2 and p‐ERK1/2 after transfection with EREG‐specific siRNAs. (f) Western blotting analysis protein levels of EREG, p‐p38, p38, ERK1/2 and p‐ERK1/2 after transfection with miR‐320b mimics, inhibitors or circFOXP1‐specific siRNAs in BxPC‐3 cells. *p < 0.05, **p < 0.01. Table S1: RNA sequences of siRNAs and shRNAs used in this article. Table S2: Sequences of primers used in this article. Table S3: Primary and secondary antibodies used in this article. Table S4: Sequences of FISH probes used in this article. Table S5: Sequences of probes used in RNA‐pulldown experiments. Table S6: Correlation between circFOXP1 expression and clinicopathologic characteristics of PDAC patients. [file JCMM-30-e71230-s001.docx]

**Supplementary Materials**

**circFOXP1 promotes pancreatic ductal adenocarcinoma progression through regulating miR-320b/EREG/MAPK axis**

**Huang et.al**


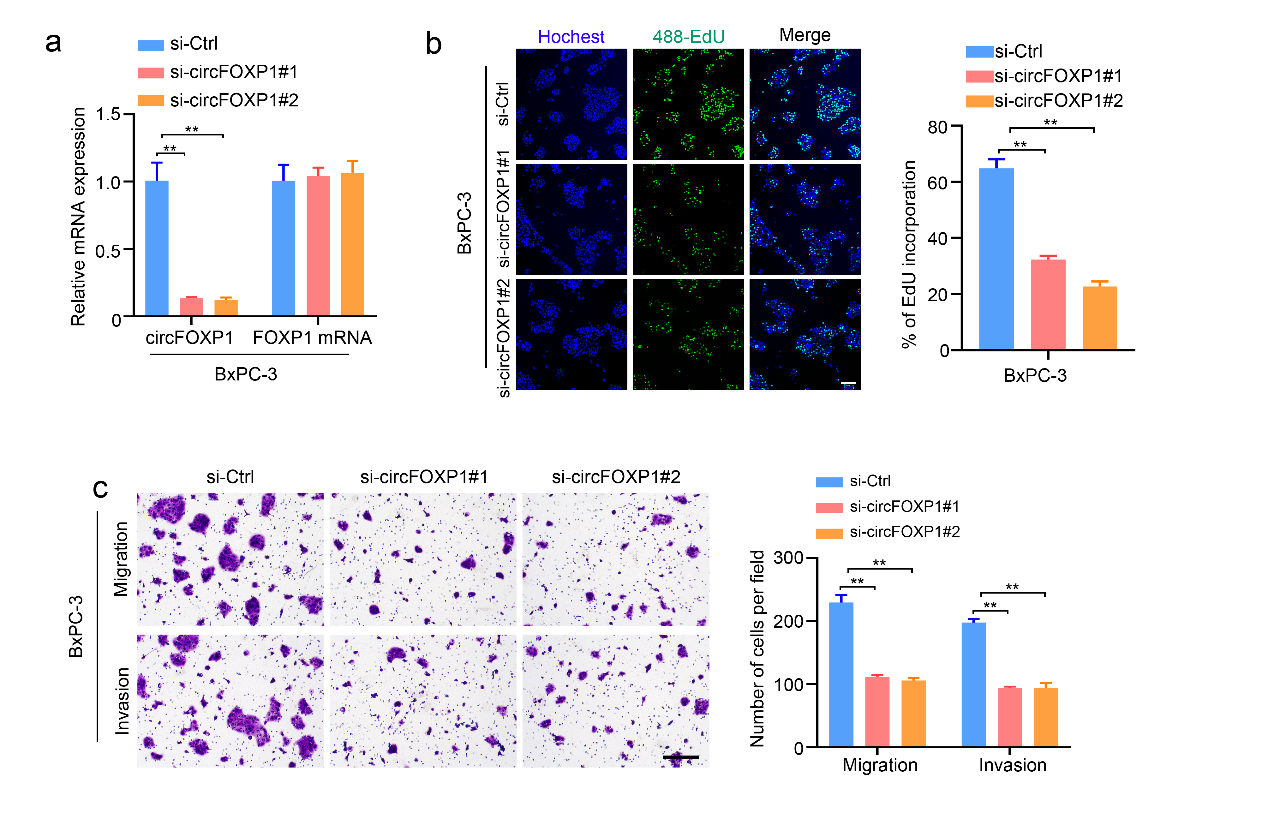


**Supplementary Figure 1. CircFOXP1 promotes proliferation, migration, and invasion of PDAC cells in vitro**. **a.** The expression of circFOXP1 and FOXP1 mRNA was assessed by qRT-PCR in BxPC-3 cells transfected with scramble or circFOXP1-specific siRNAs. **b.** EdU assays showing that knockdown of circFOXP1 inhibited the proliferation of BxPC-3 cells. **c.** The migration and invasion ability of PDAC cells were assessed using the Transwell migration and invasion assays after knocking down circFOXP1 in BxPC-3 cells. Scale bar = 100 μm. **p* < 0.05, ***p* < 0.01.


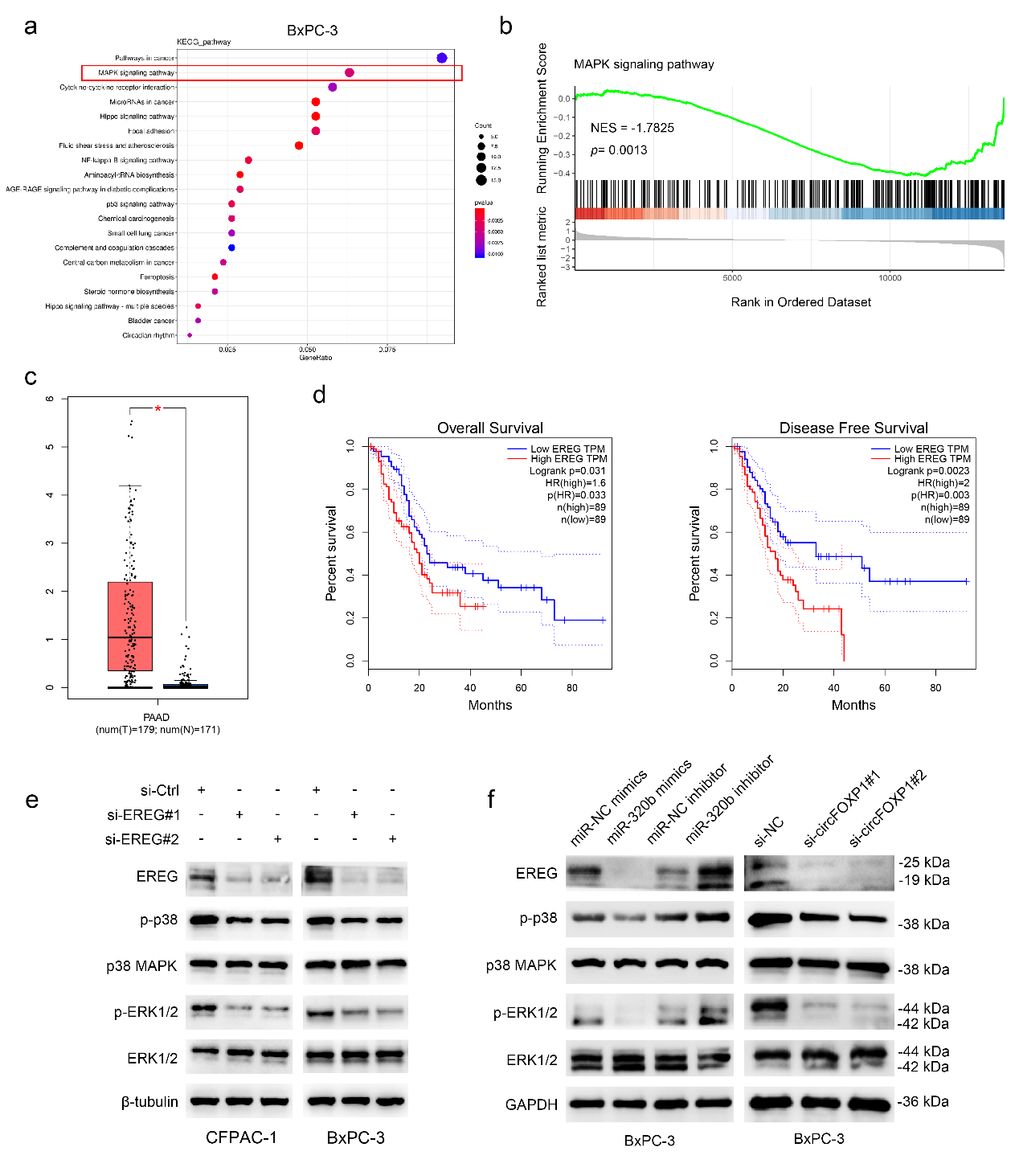


**Supplementary Figure 2. CircFOXP1 upregulates EREG expression to activate the MAPK pathway in PDAC. a.** KEGG enrichment analysis of differentially expressed genes in CFPAC-1 cells after circFOXP1 knockdown. **b.** Representative image of GSEA analysis in BxPC-3 cells showing the enrichment of the MAPK signaling pathway in BxPC-3 cells treated with sh-circFOXP1. **c.** The expression level of EREG gene was evaluated using the publicly available database GEPIA2. **d.** Using the publicly available database GEPIA2, higher expression of EREG gene was correlated with shorter overall survival and disease-free survival. **e.** Western blotting analysis protein levels of EREG, p-p38, p38, ERK1/2 and p-ERK1/2 after transfection with EREG-specific siRNAs. **f.** Western blotting analysis protein levels of EREG, p-p38, p38, ERK1/2 and p-ERK1/2 after transfection with miR-320b mimics, inhibitors or circFOXP1-specific siRNAs in BxPC-3 cells. **p* < 0.05, ***p* < 0.01.

**Supplementary Table 1. RNA sequences of siRNAs and shRNAs used in this article**

| **Target Name** | **Categories** | **Sense (5’ to 3’)** | **Antisense (5’ to 3’)** |
| --- | --- | --- | --- |
| si-Ctrl | siRNA | UUCUCCGAACGUGUCACGUTT | ACGUGACACGUUCGGAGAATT |
| si-CircFOXP1#1 | siRNA | CAAUCAUUUCUAAAGUUCCTT | GGAACUUUAGAAAUGAUUGTT |
| si-CircFOXP1#2 | siRNA | CUAAAGUUCCCGUGUCAGUTT | ACUGACACGGGAACUUUAGTT |
| si-EREG#1 | siRNA | GCUCAAGUGUCAAUAACAATT | UUGUUAUUGACACUUGAGCTT |
| sh-Ctrl | shRNA | TTCTCCGAACGTGTCACGT | |
| sh-CircFOXP1#1 | shRNA | CCGGCAATCATTTCTAAAGTTCCCTCGAGGGAACTTTAGAAATGATTGTTTTTGAATT | |
| sh-CircFOXP1#2 | shRNA | CCGGCTAAAGTTCCCGTGTCAGTCTCGAGACTGACACGGGAACTTTAGTTTTTGAATT | |

**Supplementary Table 2. Sequences of primers used in this article**

| **Target Name** | **Forward primer (5’ to 3’)** | **Reverse primer (5’ to 3’)** |
| --- | --- | --- |
| circFOXP1 divergent primer | GCACCTTCCAAGACCTCCTT | CACTGACACGGGAACTTTAGA |
| circFOXP1 convergent primer | TGACACCTCAAGTTATCACTCC | CGCTGCAAAGACAGGAGG |
| Linear FOXP1 mRNA | ATGATGCAAGAATCTGGGACTG | AGCTGGTTGTTTGTCATTCCTC |
| has-miR-370-3p | GGCCTGCTGGGGTGGAA | AGTGCAGGGTCCGAGGTATT |
| has-miR-320b | GCGAAAAGCTGGGTTGAGA | AGTGCAGGGTCCGAGGTATT |
| has-miR-93-3p | CGCGACTGCTGAGCTAGCAC | AGTGCAGGGTCCGAGGTATT |
| has-miR-1226-5p | GGGCATGCAGGCCTGG | AGTGCAGGGTCCGAGGTATT |
| has-miR-619-3p | CGGACCTGGACATGTTTGTG | AGTGCAGGGTCCGAGGTATT |
| has-miR-490-3p | CGCAACCTGGAGGACTCC | AGTGCAGGGTCCGAGGTATT |
| has-miR-127-5p | CGCTGAAGCTCAGAGGGC | AGTGCAGGGTCCGAGGTATT |
| has-miR-558 | GCGCGTGAGCTGCTGTAC | AGTGCAGGGTCCGAGGTATT |
| has-miR-20a-3p | CGCGACTGCATTATGAGCAC | AGTGCAGGGTCCGAGGTATT |
| HERPUD1 | ATGGAGTCCGAGACCGAAC | TTGGTGATCCAACAACAGCTT |
| EREG | GTGATTCCATCATGTATCCCAGG | GCCATTCATGTCAGAGCTACACT |
| SPOPL | TACTGGTCCCATAGCAGAAAGC | CCCATTTCCTCTCGACAAAAACT |
| TIPARP | AGAACGAGTGGTTCCAATCCA | TGGGTGCAAAAGATCAGTCTG |
| MSANTD4 | GAAGGCCAACATTAAGCTGGT | CTCTTCAGTGAGAGAGTCATCCA |
| YOD1 | ATGTTTGGCCCCGCTAAAGG | CGGTGATGGCGGCAATTTG |
| GAPDH | GGAGCGAGATCCCTCCAAAAT | GGCTGTTGTCATACTTCTCATGG |

**Supplementary Table 3. Primary and secondary antibodies used in this article**

| **Antibody** | **Species** | **Type** | **Dilution** | **Sequence (5’ to 3’)** |
| --- | --- | --- | --- | --- |
| GAPDH | Mouse | Monoclonal | 1:1000 (WB) | Ray Antibody |
| Ki-67 | Rabbit | Polyclonal | 1:300 (IHC) | Abcam |
| EREG | Rabbit | Polyclonal | 1:300 (IHC);  1:1000 (WB) | Abcam |
| p38 MAPK | Rabbit | Monoclonal | 1:1000 (WB) | Cell Signaling Technology |
| p-p38 MAPK | Rabbit | Monoclonal | 1:1000 (WB) | Cell Signaling Technology |
| ERK1/2 | Rabbit | Monoclonal | 1:1000 (WB) | Cell Signaling Technology |
| p-ERK1/2 | Rabbit | Monoclonal | 1:1000 (WB) | Cell Signaling Technology |

**Supplementary Table 4. Sequences of FISH probes used in this article**

| **Target Name** | **Sequence (5’ to 3’)** |
| --- | --- |
| circFOXP1 | CCACUGACACGGGAACUUUAGAAAUGAUUGG |
| miR-320b | TTGCCCTCTCAACCCAGCTTTT |

**Supplementary Table 5. Sequences of probes used in RNA-pulldown experiments**

| **Target Name** | **Sequence (5’ to 3’)** |
| --- | --- |
| Negative control | UUCUCCGAACGUGUCACGUTT |
| circFOXP1 | CCACUGACACGGGAACUUUAGAAAUGAUUGG |

**Supplementary Table 6. Correlation between circFOXP1 expression and clinicopathologic characteristics of PDAC patients**

| **Characteristics** | **No. of cases** | **circFOXP1 expression level** | | |
| --- | --- | --- | --- | --- |
|  |  | **Low** | **High** | ***p*-value^A^** |
| **Total cases** | 160 | 80 | 80 |  |
| **Gender** |  |  |  | 0.872 |
| Male | 93 | 47 | 46 |  |
| Female | 67 | 33 | 34 |  |
| **Age** |  |  |  | 0.327 |
| ≤60 | 60 | 33 | 27 |  |
| ＞60 | 100 | 47 | 53 |  |
| **Differentiation** |  |  |  | 0.599 |
| Poor | 30 | 13 | 17 |  |
| Moderate | 98 | 52 | 46 |  |
| Well | 32 | 15 | 17 |  |
| **T stage** |  |  |  | 0.633 |
| T1-2 | 89 | 46 | 43 |  |
| T3-4 | 71 | 34 | 37 |  |
| **Lymphatic metastasis** |  |  |  | 0.075 |
| Negative | 63 | 37 | 26 |  |
| Positive | 97 | 43 | 54 |  |
| **TNM stage** |  |  |  | **0.044^*^** |
| Stage I | 34 | 22 | 12 |  |
| Stage II | 82 | 42 | 40 |  |
| Stage III | 44 | 16 | 28 |  |

Abbreviations: No. of cases = number of cases; T stage = tumor stage; TNM stage = tumor node metastasis stage.

^A^ Chi-square test, ^*^ *p* <0.05, ^**^ *p* <0.01.
